# Voice Congruency Facilitates Word Recognition

**DOI:** 10.1371/journal.pone.0058778

**Published:** 2013-03-20

**Authors:** Sandra Campeanu, Fergus I. M. Craik, Claude Alain

**Affiliations:** 1 Rotman Research Institute, Baycrest Centre for Geriatric Care, Toronto, Ontario, Canada; 2 Department of Psychology, University of Toronto, Toronto, Ontario, Canada; University of Barcelona, Spain

## Abstract

Behavioral studies of spoken word memory have shown that context congruency facilitates both word and source recognition, though the level at which context exerts its influence remains equivocal. We measured event-related potentials (ERPs) while participants performed both types of recognition task with words spoken in four voices. Two voice parameters (i.e., gender and accent) varied between speakers, with the possibility that none, one or two of these parameters was congruent between study and test. Results indicated that reinstating the study voice at test facilitated both word and source recognition, compared to similar or no context congruency at test. Behavioral effects were paralleled by two ERP modulations. First, in the word recognition test, the left parietal old/new effect showed a positive deflection reflective of context congruency between study and test words. Namely, the same speaker condition provided the most positive deflection of all correctly identified old words. In the source recognition test, a right frontal positivity was found for the same speaker condition compared to the different speaker conditions, regardless of response success. Taken together, the results of this study suggest that the benefit of context congruency is reflected behaviorally and in ERP modulations traditionally associated with recognition memory.

## Introduction

There is considerable evidence that reinstating the initial auditory context at the time of test aids memory for spoken words [1]–[4]. For instance, in a continuous recognition task, participants recognized spoken words more quickly and accurately when they were re-presented in the same voice at test [1]. This same-voice advantage might be limited to the identical speaker, since Palmeri et al. [2] found that similar voices (i.e., same gender) do not yield a significant facilitation. Pisoni [5] posited a parallel episodic memory system, perceptual and implicit, which encodes information about speaker voice (e.g., gender and dialect) with memory for the item itself. This suggestion implies a benefit for context congruency in memory for spoken words, and more generally is in line with the principle of transfer-appropriate processing [6].

Goldinger [4] also found a same-voice advantage in word recognition. However, he also noted better recognition for words that were re-presented in a different voice of the same gender than for those re-presented in a different voice of a different gender, suggesting some degree of generalization in the effects of voice congruency. That is, the identical voice condition yielded the best word recognition, followed by the similar voice condition (same gender), and lastly the dissimilar voice condition (different gender). This is in contrast with Palmeri et al.’s [2] finding, which implied a need for identical voice at test to facilitate recognition. Therefore it remains unclear whether there is an additive benefit from reinstating similar voice parameters, or if voice specificity is necessary to facilitate recognition memory. The present work investigates memory when two voice parameters are varied between study and test, in an effort to shed some light on this question. It is important to note that some studies have yielded no same-voice benefit for word memory [7]–[11]; possible reasons for this difference include longer retention intervals, participants’ increased reliance on conceptual rather than perceptual encoding, presence of distracter tasks between study and test, and inclusion of a voice familiarization phase. Some or all of these factors may have confounded a straight comparison of voice effects as we intend to study them.

The literature concerning source (speaker) memory is also mixed. Palmeri et al. [2] asked their participants to identify whether a test word is new, old with the same voice as at study, or old with a different voice than at study and found a same-speaker benefit for source memory. In other studies, participants were asked to first identify a test word as old or new and then subsequently determine whether the speaker’s voice for old test words was the same as, or different than, the study voice [1], [8]. In the Craik and Kirsner study [1], no same-voice benefit for source recognition was observed. However, it should be noted that the lack of same-voice source memory facilitation in that study might be related to the fact that only two voices (male, female) were used, thereby reducing the source judgment to a simple gender classification task.

In the present study, we seek to extend previous work by assessing how two voice parameters (two aspects of a source) interact in both word and speaker memory. Prior studies used voice pitch (e.g., gender) as the source characteristic of choice. In an effort to investigate the effect of multiple source characteristics on word and source recognition, we examine the benefits of reinstating the same speaker when both pitch (large difference between genders) and accent (Chinese or Canadian) vary.

Moreover, by using scalp recordings of event-related potentials (ERPs), the present work aims to clarify when and over which scalp regions context congruency exerts its influence on word and source memory. Performance during word and source recognition tasks is generally associated with a left parietal old/new ERP effect at retrieval. This modulation, beginning around 400 ms post-stimulus and lasting several hundred milliseconds [12]–[16], quantitatively indexes recollection and its amplitude reflects how well the word is remembered. In addition, though some work suggests that speaker voice is automatically encoded with the word [17]–[18], more recent ERP findings fail to support this position [19]. Senkfor and Van Petten [19] tested memory for words and speakers, specifically testing only speakers for old words in the source task. Based on observed ERP latencies, including a source-related positivity over the right scalp region starting at 800 ms after word onset, they concluded that voice information was retrieved after word information, suggesting a hierarchical system. This contrasts with Pisoni’s parallel system [5], which implies the possibility of simultaneous retrieval. However, it is important to note that the study by Senkfor and Van Petten emphasized semantic encoding. It might be that placing the emphasis on perceptual encoding could facilitate an earlier, perhaps more automatic retrieval of speaker in the source task. Indeed, research has shown that voice congruency effects are more pronounced when perceptual encoding is emphasized [4].

In the present study, we investigate the effects of context congruency on word and source recognition, wherein context might be reinstated at test, might vary in either gender or accent while remaining congruent on the other voice parameter or might largely vary on both voice parameters. Based on previous work, we predict a same-speaker (reinstatement) advantage in both the word recognition test and the source (speaker) memory test, indicating either specificity for voice-word binding or a quantitative benefit of context congruency at test. While a gender or accent congruency effect is possible, no clear hypothesis can be stated (e.g., Goldinger [4] vs. Palmeri et al [2]). However, given that Goldinger assessed inter-voice similarity prior to testing voice effects in recognition by using a scaling matrix based on relative pitch (of male and female voices), his finding of a gender effect seems perhaps more convincing than Palmeri’s null gender finding in the item recognition test. Such a partial congruency facilitation effect would fall in line with the hypothesis of a graded voice congruency benefit, but we can make no prediction about pitch versus accent as a congruency cue.

Two ERP modulations are also expected. Given that the parietal old/new effect is generally thought to reflect a quantitative measure of recollection, one could anticipate that the old/new effect will be larger when test words are spoken by the same voice as at study (reinstatement) than when spoken by a different voice. However, it is unclear whether the old/new effect would also reflect partial voice congruency between study and test. In addition, we predict a right frontal deflection in the source task at a somewhat earlier latency than the one found by Senkfor and Van Petten [19]. Moreover, though this modulation has typically been investigated post-retrieval, recent work by Hayama et al. [20] has indicated that the right frontal deflection might actually index decision/judgment processes. If this is the case, then the modulation may not be subsequent to item recognition, but may reflect processes involved in source judgments. If this analysis is correct, we would not expect a right frontal effect in the word test (where a source judgment is not required), but would expect one in the speaker test (where item recognition is not tested).

## Methods

### Participants

Twenty-two participants provided written informed consent according to the guidelines set out by the Baycrest Centre and the University of Toronto. One participant did not complete the experiment because their pure-tone threshold fell out of the normal range in the left ear. EEG data from two participants were excluded because of excessive muscle artifacts and/or eye movements during recording. Lastly, three participants were excluded because of insufficient number of trials per condition. The final sample of 16 participants comprised four males and twelve females aged between 19 and 32 (M  =  25 ± 4.3 years); they all had English as their first language, were right-handed and had pure-tone thresholds within normal limits for frequencies ranging from 250 to 8000 Hz (both ears).

### Stimuli and Design

The word list consisted of 336 high-frequency (30+ from Kucera & Francis, [21]), two-syllable nouns, taken from the MRC psycholinguistic database [22]. All words were recorded by four speakers - one native-English female, one native-English male, one Chinese-accented female and one Chinese-accented male – in continuous streams (32,000 Hz sampling rate, mono, 16-bit resolution). The native English-speaking female was 31 years old at the time of recording and the native English-speaking male was 27 years old. The Chinese-accented female was 57 years old and had learned English at 27 years of age. The Chinese-accented male was 44 years old and had learned English at 19 years of age. With a Shure KSM44 microphone and an USBPre preamplifier with digitizer, speakers recorded the words using Adobe Audition 1.5 on a Dell laptop. All words were then spliced into individual files, using a matlab (version 5.3) script, and their amplitudes were normalized to a standard dB level (the average of all words). The matlab script used to splice the words specified thresholds, duration and pre-stimulus intervals, which could be varied as necessary for different groups of words. Words were inspected for background noise, clipping, duration (1 second) and clarity. Editing was done as required, either manually or using batch files. To check for intelligibility, two young adults with English as a first language each listened to three blocks of words and indicated where word intelligibility would be an issue. Reshuffling and processing of new words occurred as necessary to ensure that potential participants (young adults with English as a first language) would have no trouble understanding the words spoken. Once all words were judged adequate, they were normalized to their average loudness, at -32.44dB.

The experiment was programmed using Presentation software (version 11.0). Words (as *.wav files) were converted to analogue using a computer soundcard (44,100 Hz sampling rate, stereo, 16-bit resolution). The analogue output was fed into a 10 kHz filter (Tucker Davis Technologies (TDT, Alachua, FL), FT6-2), and then to a GSI 61 audiometer. Stimuli were presented binaurally through insert earphones (EAR-TONE 3a), at 70dB SPL. The words were divided into six independent blocks and balanced such that words beginning with each letter were equally distributed into the six blocks. The six blocks, which were identical in structure, consisted of a study phase of 32 words, plus 4 buffer words on each end, and two recognition tests. There were 8 study words in each of the 4 voices. The word recognition task used 16 studied words and 16 new words and asked participants to give an old/new judgment for each word. In terms of voices, 4 words that were originally spoken in each voice at study were presented during the word recognition task. Participants were asked to judge a word as “old” if they had heard it earlier, irrespective of whether the test word was presented in the same or a different voice as at study. However, the number of words in each voice was also balanced in each word recognition test. The second task (source recognition) used the other 16 studied words only, and asked participants for a yes/no answer as to whether the speaker for that word at test was the same as during study. Again, each source recognition test was balanced for the number of words presented in each voice. In each block, there were four ‘same speaker’ conditions for each test, one for each voice. This gave a total of 24 same speaker trials per participant. There were also four ‘different gender/same accent’ and four ‘same gender/different accent’ conditions, as well as four ‘different gender/different accent’ conditions, in each test of each block. This made each voice congruency condition possible, with each voice, in each test and each block. Participants were given mandatory 2-minute breaks between blocks.

Each scenario began with a warning sound (1.5 seconds duration) to alert participants that the word list was about to begin. The first word was presented 2 seconds after the cue, and subsequently the stimulus onset asynchrony (SOA) between words (in the study phase) was 3 seconds. In the encoding phase, participants were instructed to pay attention to both the words and the speakers of each word. They were told that there would be two subsequent tests, one for word recognition and another for source recognition. Since the word and source recognition tasks used different words, counterbalancing order of the tests was not needed. Instead, we felt that it was important to maintain a consistent test order so that duration between study and each test was the same for all participants. Therefore, we presented the word recognition test first during each block. All participants received the same test lists. During the test phases, participants responded to the old/new and same speaker/different speaker judgments in a self-paced manner. There was no visual instruction screen in either the study or the test phases; stimuli were strictly auditory. Time between the end of the study phase and the beginning of the word recognition test was approximately 30 s – 1 min. Time between the end of the word recognition test and the beginning of the source recognition test was only a few seconds, but again, studied words did not overlap between tests. Responses were made on a keyboard using the right hand (the dominant hand since all participants were right-handed), but we did not control for which fingers were used.

### Behavioral Analysis

For the word recognition task, a sensitivity measure was calculated for the four possible voice conditions – same speaker, same gender/different accent, different gender/same accent, different gender/different accent – by subtracting false alarms from hit rates. The false alarm rate used was a common rate based on the proportion of new words incorrectly judged as old, for each participant.

For the source memory test, a hit rate was used for the same speaker condition. This hit rate was compared to the chance level for the same speaker condition (0.25) in a one-sample t-test. For the three different speaker conditions in the source memory test, correct responses were represented by correct rejections, given that participants were asked to simply respond “same” or “different” rather than distinguishing between the different speaker conditions. For this reason, false alarm rates were compared for these three conditions.

### Electrophysiological Recording and Analysis

The electroencephalogram (EEG) was digitized continuously (sampling rate 500 Hz; bandpass of 0.05–100 Hz) during the study phase and the test phases using NeuroScan Synamps2 (Compumedics, El Paso, TX, USA). The ERPs were sampled at 64 scalp locations that include electrodes placed at the outer canthi and at the inferior orbits to monitor eye movements. During recording, all electrodes were referenced to the Cz electrode; for off-line data analysis, they were re-referenced to an average reference. The analysis epoch consisted of 200 ms of pre-stimulus activity and 1300 ms of post-stimulus activity.

For each participant, a set of ocular movements was obtained before and after the experiment [23]. A matlab program was used to calculate averaged eye movements for both lateral and vertical eye movements as well as for eye-blinks. A principal component analysis of these averaged recordings provided a set of components that best explained the eye movements. The scalp projections of these components were then subtracted from the experimental ERPs to minimize ocular contamination, using Brain Electrical Source Analysis (BESA 5.2).

After correcting for eye movements, all experimental files for each participant were then scanned for artifacts; epochs including deflections exceeding 130 µV were marked and excluded from the analysis. The remaining epochs were averaged according to electrode position and trial type, using BESA 5.2. Each average was baseline-corrected with respect to the pre-stimulus interval and digitally low-pass filtered at 20 Hz (zero phase, 24 dB/oct), using BESA software.

As previously mentioned, files were collapsed across the six blocks for each participant; weighted combinations for each condition per participant were made. Group average files were then made by combining all participants’ blocks in an unweighted fashion. The parietal old/new effect has been consistently found in the literature to index recollection when old/new judgments are required, as is the case in the present study. Tendolkar et al. [14] described that “[t]he first old/new effect identified during tests of recognition memory onsets approximately 400 ms post-stimulus, typically lasts around 400–600 ms, and is largest in amplitude over left temporo-parietal scalp electrodes” (p.236). To both correspond with that expected latency range and to surround the observed peak of the deflection in the present study, the modulation was quantified here at the 700–900 ms interval, over P5, P3, PO3 and P1 on the left side and P6, P4, PO4 and P2 on the right side. Electrodes were chosen to correspond to scalp areas analyzed in previous, relevant studies [12]–[15]. Mean amplitude measurements were exported and analyzed using repeated measure ANOVAs with trial type as the within-subject factor.

The right frontal effect is traditionally described for studies where a source judgment follows an old/new judgment. Since this study used a different methodology, we could not predict a clear time window based on the literature. As such, the right frontal deflection predicted for the source test was quantified using more objective pairwise permutation tests, using BESA Statistics 1.0. Comparisons were made between the same speaker voice condition and the different speaker voice condition, regardless of response success, from 100 ms to 1300 ms post-stimulus over the entire scalp. This two-stage analysis first computes a series of t-tests that compare the ERP amplitude between the two conditions at every time point. This identifies clusters when the ERPs differ between the conditions. In the second stage of this analysis, permutation tests are performed on these clusters. The permutation test uses a bootstrapping technique to determine the probability values for differences between conditions in each cluster. The final probability value computed is based on the proportion of permutations that are significant for each cluster, and implicitly corrects for multiple comparisons. In the current analysis, we used a cluster alpha of 0.05, one thousand permutations and clusters defined using a channel distance of 4 cm, which resulted in an average of 3.125 neighbors per channel. The literature points to a right frontal modulation before 800 ms (see introduction and [19]). Based on this, we examined significance in the right frontal area between 100 ms–900 ms.

In the word recognition test, the parietal old/new effect was measured using correct trials only; hence, the mean number of correct trials used in each condition was 17.8 (range 11–22, s.d. 2.63). In the source recognition test, the mean number of correct trials used in the same speaker condition was 17.1 (range 14–20, s.d. 2.2). Since the different gender/same accent, same gender/different accent, and different gender/different accent conditions all resulted in the same response, “different voice”, in the source recognition test, they were combined together (mean number of trials per condition  =  13.5; range 5–21, s.d. 3.8).

## Results

### Word Recognition Test

#### Behavioral Results

**Figure 1:**
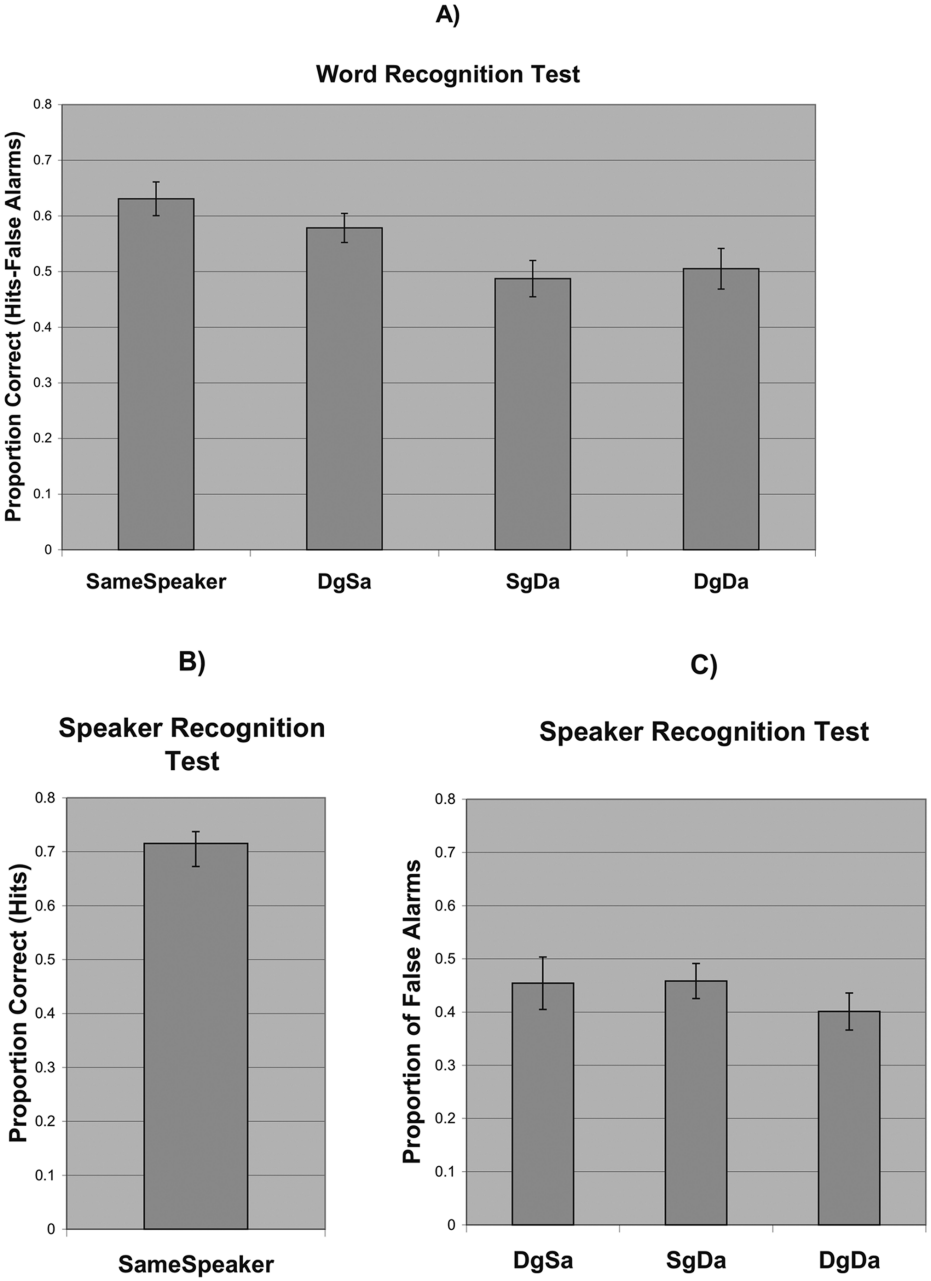
a) Accuracy for the Word Recognition Test, b) Accuracy for the Source recognition Test, c) False Alarms for the Source recognition Test. Note that DgSa  =  different gender/same accent condition, SgDa  =  same gender/different accent condition and DgDa  =  different gender/different accent condition. Error bars represent standard error.

Figure 1a shows the group mean accuracy for all four conditions in the word recognition test. The ANOVA yielded a main effect of voice congruency (F(3,45) = 11.41, p<0.001, η^2^  =  0.43), with pairwise comparisons indicating that performance in the same speaker condition was superior to all three different speaker conditions (p<0.05). In addition, performance in the different gender/same accent condition was significantly better than in the same gender/different accent condition and the different gender/different accent condition (p<0.05). Consistent with this, a preliminary analysis using gender and accent as independent factors found only a main effect of accent in the word test (F(1,60) = 11.74, p = 0.001, η^2^  =  0.164).

#### ERP Results

**Figure 2:**
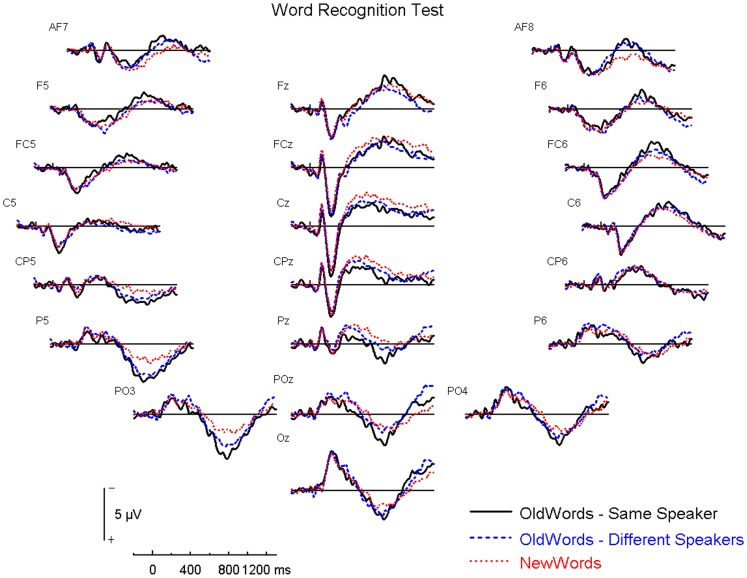
Word Recognition Test – Panel of Electrodes. SS  =  same speaker; DD  =  different gender/different accent; NW  =  new word.

Figure 2 shows the ERP results for the word recognition test at various electrode sites over the entire scalp.

##### 1) Left Parietal Old/New Effect

In the word recognition test there was an increased positivity for correctly identified old words as compared to correctly identified new words, which peaked at around 750 ms post-stimulus. The ANOVA on the mean amplitude for the 700–900 ms interval over bilateral parietal regions yielded a significant main effect of trial type, F(1,15) = 11.70, p = 0.004, η^2^ = 0.44, with old words producing a significantly more positive deflection than new words. There was also a main effect of hemisphere, F(1,15) = 4.96, p = 0.042, η^2^ = 0.25, with a significantly more positive deflection over the left hemisphere. Lastly, there was also a significant trial type x hemisphere interaction, F(1,15) = 24.54, p<0.001, η^2^ = 0.62. Due to the hemispheric effect, which coincides with the literature in naming this deflection as the left parietal old/new effect, we restricted further analyses to the left hemisphere only.

**Figure 3:**
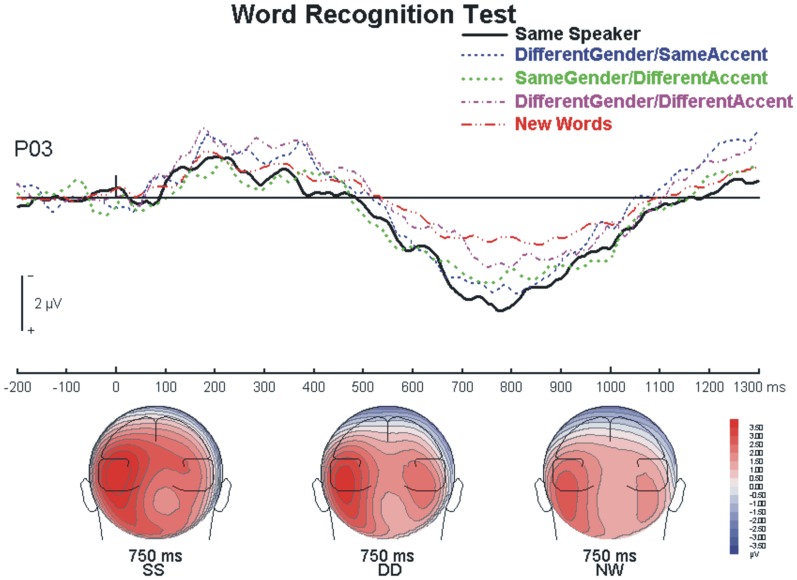
Left Parietal Old/New Effect. Recorded over the left parieto-occipital site (i.e., PO3) during the Word Recognition Test, showing all five conditions. SS  =  same speaker; DD  =  different gender/different accent; NW  =  new word.

We then analyzed the left parietal old/new deflection over P5, P3, PO3 and P1 at 700–900 ms for all five trial types – same speaker, different gender/same accent, same gender/different accent, different gender/different accent and new words. There was a main effect of trial type, F(4,60) = 4.55, p = 0.011, η^2^  =  0.23, with pairwise comparisons indicating that the same speaker, the different gender/same accent and the same gender/different accent conditions were each more positive than the new word condition (p<0.05). The correctly identified new words had the smallest positivity (see Figure 3).

##### 2) Right Frontal Effect

Research by Hayama et al. [20] indicated that right frontal deflections traditionally associated with post-retrieval work are actually indicative of decision/judgment processes rather than post-episodic source retrieval processing. To that end, we analyzed the item recognition data and found no late right frontal effect, indicating support for the decision/judgment hypothesis over the source retrieval hypothesis. Since no source judgment was required in the word recognition test, it is not surprising that we found no late right frontal effect here.

### Source Recognition Test

#### Behavioral Results

For the source recognition test, the same speaker performance measure is shown in Figure 1b along with the false alarm rates taken to measure performance in the other three conditions (Figure 1c). Since participants were asked to make a “same” vs. “different” speaker judgment, the analysis of hit rates for the three different speaker conditions would not take into account any bias associated with simply responding “different” without having to clarify which type of different speaker condition was present. Therefore, we restricted our analyses to the hit rate for the same speaker condition and to the false alarm rates for the three different speaker conditions.

Two analyses were performed on the accuracy data from the source memory test. For the same speaker condition a one-sample t-test was conducted to see if the group mean was significantly above chance (25%, since ¼ of the trials were same speaker trials). Accuracy for the same speaker condition was significantly above chance (t(15) = 21.07, p<0.001, r^2^  =  0.97). Next, we compared the false alarm rates for the other three (different) speaker conditions to see if there was a bias. These were analyzed in a repeated-measures ANOVA. Even though the different gender/different accent false alarm rate was somewhat smaller than the other two, this effect was not significant, (F(2,30) = 1.32, p>0.2). Therefore, there appeared to be no bias between the three different speaker conditions in the source memory test.

#### ERP Findings

**Figure 4:**
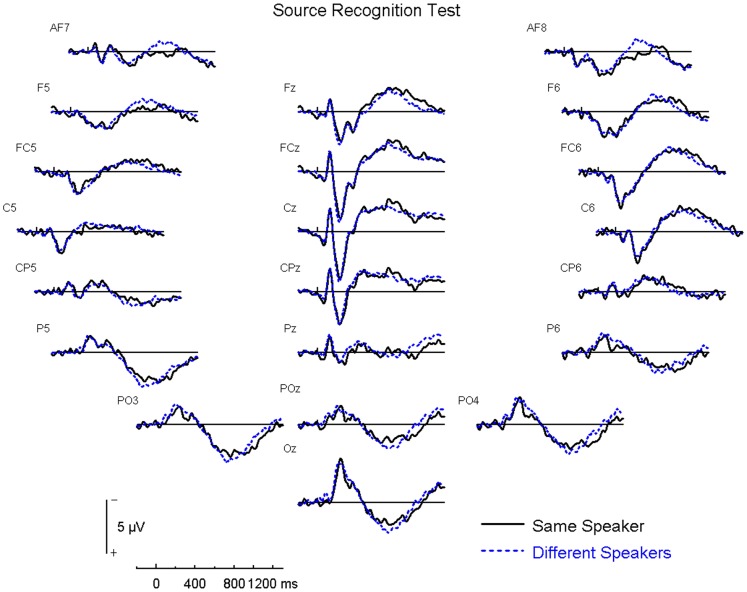
Source Recognition Test – Panel of Electrodes. The three different speaker conditions are collapsed into the “Different Speakers” condition shown here.

Figure 4 shows the ERP results for the source recognition test at various electrode sites over the entire scalp.

##### 1) Left Parietal Old/New Effect

Since all words presented in the source recognition test were old words, and participants were aware of this, it is not surprising that there was a consistent positivity over parietal regions for all words.

##### 2) Right Frontal Effect

**Figure 5:**
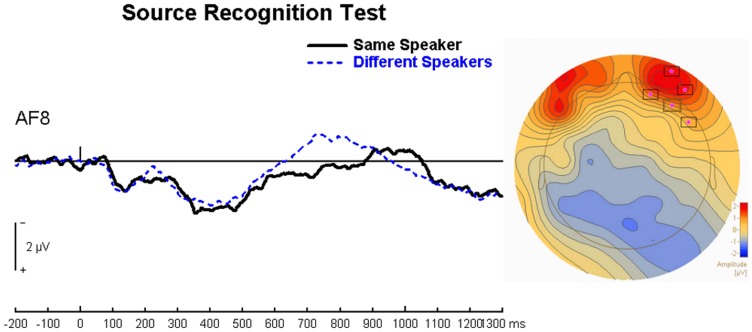
Right Frontal Effect. a) Waveform recorded over AF8, showing the same speaker condition versus the collapsed different speaker condition, b) Scalp topography over the top of the head, showing the significant cluster over right frontal regions found using BESA Statistics 1.0, in the Source Recognition Test.

The right frontal effect was quantified in the source recognition test. Since the hypothesized right frontal effect was not predicted at a specific time window, we conducted an analysis using BESA Statistics 1.0, over all scalp regions for 100–1300 ms post-stimulus, and were particularly interested in significant modulations found over the right frontal area between 100–900 ms. Since previous research has shown that the right frontal effect is found for both correct and incorrect source judgment trials (19), we compared all same speaker trials vs. all different speaker trials, regardless of response success. The results of this cluster analysis produced a significant difference between same speaker and different speaker conditions over right frontal electrodes (FP2, AF8 and F8), which peaked at 722 ms (p = 0.016). The same speaker condition produced a significantly more positive deflection than the collapsed different speaker condition (Figure 5).

We also conducted a systematic univariate analysis, using ANOVAs at 100 ms intervals, beginning at 100 ms post-stimulus, bilaterally over AF7, FP1 and AF8, FP2. The windows that produced a significant main effect of voice condition were 700–800 ms, F(1,15) = 6.13, p<0.05 and 800–900 ms, F(1,15) = 5.54, p<0.05. Surprisingly, the ANOVAs indicated a bilateral modulation, showing no hemispheric effect.

## Discussion

The purpose of the present study was to assess the impact of reinstating speaker voice context at test on both word and source memory, and to identify memory-related ERPs that reflect a potential voice congruency benefit.

### Behavioral Findings

In the present study, participants were most likely to remember a word if it was presented with the same speaker voice at study and at test. This finding is consistent with Pisoni’s [5] notion of a parallel memory system that encodes speaker voice with word memory, and with prior behavioral research showing that reinstating the study voice at test provides a performance benefit both in word (e.g., [1]) and source [2] memory. In addition, we found facilitation in word recognition when the accent of the speaker was congruent with the original presentation, compared to reinstating gender or neither characteristic of voice. Though this provides some support to prior research indicating a context congruency benefit in word recognition (see [4]), further research is needed to clarify why the effect was restricted to accent. Our findings may be related to the fact that the words used in the present experiment were recorded individually, and therefore were not identical on any one parameter (accent or gender) in the conditions when that parameter was said to be maintained. That is, the voice conditions represented a categorical distinction rather than a quantitative gradient. Alternatively, it may be that certain voice characteristics are more salient than others.

### ERP findings

There were two expected ERP deflections. The left parietal effect in the word recognition test produced a positivity gradient. Correctly identified old words where voice was also reinstated provided the most positive deflection. As the voice at test became less similar to the study voice, the correctly identified old words produced a weaker parietal positivity. Lastly, the correctly identified new words were the least positive of the conditions. This finding is somewhat surprising in light of work done by Schloerscheidt and Rugg [24], which indicated that the old/new parietal effect was the same for within and across format hits when verbal test stimuli were used, thus showing the irrelevance of surface form for this effect. However, their results do not speak to the effect of manipulating parameters within one of the formats, on this modulation. A possible explanation for the observed gradient is that, because only correct trials were used in the present comparison, the positivity gradient might reflect an implicit benefit of reinstating the speaker’s voice at test. This would then fall in line with findings by Wilding et al. [12] and Wilding and Rugg [13], who found that recollection was associated with greater positivity when context was recollected as well as the word itself. Moreover, our result is also in line with the description by Tendolkar [14], that this well-known effect is actually two separate effects that overlap – one that is constant for recollection and another that varies based on strength of the memory.

The second predicted ERP modulation was a late right frontal effect traditionally associated with source processing. Though this modulation has typically been thought to investigate post-retrieval processing, recent work by Hayama et al. [20] has indicated that the deflection might actually index decision/judgment processes. Indeed, the word recognition test indicated no late right frontal ERP effect, lending support to Hayama et al.’s theory. Therefore, we investigated the right frontal effect in the source test only. We compared trials across the two voice conditions, same speaker and different speaker, regardless of response success. Trials were therefore classified by voice congruency type rather than by response type. This falls in line with evidence from previous studies that show a right frontal effect for correct as well as incorrect source judgments (e.g. [19], see [20] for a brief summary). The finding that the same speaker condition produces a more positive deflection than the different speaker collapsed condition, peaking at 722 ms over right frontal sites, indicates a difference between voice congruency conditions regardless of response success. Given that the same speaker condition involves specificity in the binding of word and voice while the different speaker conditions imply less demanding (and specific) judgments, we suggest that the more positive deflection associated with same speaker trials might reflect greater monitoring demands in this more stringent voice condition. However, this suggested explanation requires future research to confirm our findings and directly test this theory.

## Conclusions

Behavioral results indicate that voice congruency at test facilitates both word memory and source memory. These behavioral effects were paralleled by two expected ERP modulations. In the word recognition test, the same speaker conditions corresponded to the most positive left parietal old/new deflection. Since this condition had the most context congruency between study and test (i.e. reinstatement), it follows that recollection was the strongest in recognition for same speaker trials. In the source recognition test, a data-driven analysis indicated a right frontal positive deflection for the same speaker trials compared with the different speaker trials, regardless of response success. This deflection likely indexes some aspect of decision/judgment processes.
