# Correction: Voice Congruency Facilitates Word Recognition

**DOI:** 10.1371/journal.pone.0111884

**Published:** 2014-10-22

**Authors:** The PLOS ONE Staff

There are a number of errors in the legend for Figure 2, “Word Recognition Test – Panel of Electrodes.” Please see the complete, corrected Figure 2 here.

**Figure 2:**
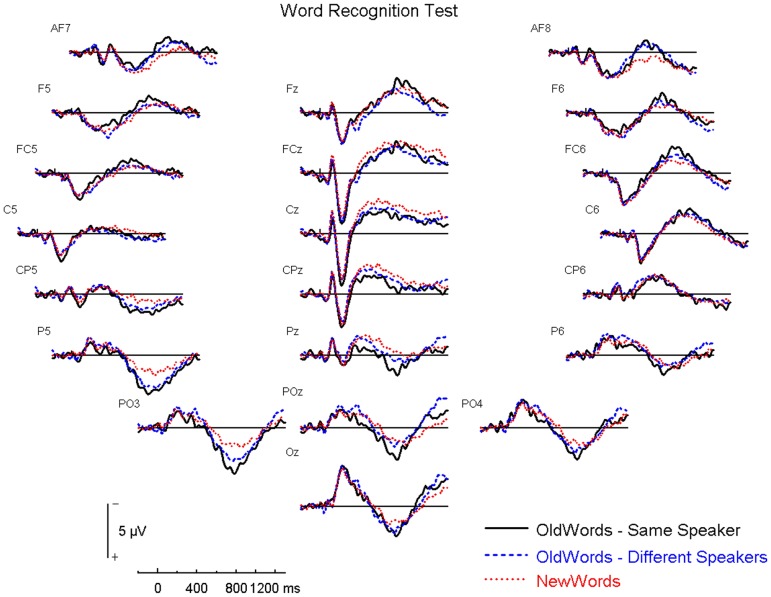
Word Recognition Test – Panel of Electrodes. The three different speaker conditions are collapsed into the “OldWords - Different Speakers” condition shown here.

There is an error in the sixth sentence of the fifth paragraph of the Introduction. The correct sentence is: Based on observed ERP latencies, including a source-related positivity over the prefrontal scalp region starting at 800 ms after word onset, they concluded that voice information was retrieved after word information, suggesting a hierarchical system.

There is an error in the fourth and fifth sentences of the last paragraph of the Introduction. The correct sentence is: In addition, we predict a late frontal deflection in the source task at a somewhat earlier latency than the one found by Senkfor and Van Petten [19]. Moreover, though this modulation has typically been investigated post-retrieval, recent work by Hayama et al. [20] has indicated that the traditionally right-lateralized frontal deflection might actually index decision/judgment processes.
